# Comparison of two validated evidence‐based medicine assessments: Do they correlate?

**DOI:** 10.1002/aet2.10831

**Published:** 2022-12-20

**Authors:** Adam Kenney, Catherine Yu, Ariel Sena, Naila Ghafoor, Shannon Moffett

**Affiliations:** ^1^ Englewood Health Englewood New Jersey USA; ^2^ Department of Emergency Medicine Rutgers New Jersey Medical School Newark New Jersey USA; ^3^ Department of Emergency Medicine University of California Los Angeles Los Angeles California USA

**Keywords:** curriculum evaluation, evidence‐based medicine, medical education research

## Abstract

Evidence‐based medicine (EBM) has been defined as a process involving five actions: asking, acquiring, appraising, applying, and assessing. Several attempts have been made to create and validate tools to assess EBM aptitude. The newest testing instrument, the ACE tool, which is a 15‐question true/false exam, has not been directly compared to the more established Fresno test, which is composed of 12 in‐depth short‐answer questions. Although both were designed to test Steps 1–4 of the five‐step EBM process, it is unclear whether they examine the same things or whether one is superior. To our knowledge there is not a widely used standard for EBM assessment despite the broad requirements for inclusion of EBM in both undergraduate and graduate medical education.

**Hypotheses:**

It was hypothesized that these instruments do not correlate between one another, based on inherent differences between them, including assessment format, grading method, and scoring range. The authors sought to examine whether a correlation between the results of these two instruments exists in a population of U.S. medical students.

**Methods:**

A retrospective cohort study of 158 fourth‐year U.S. medical students in academic year 2018–2019 was conducted. All students were exposed to a focused EBM curriculum, consisting of three guided discussions of separate journal articles clinically relevant to the practice of emergency medicine. Outcomes measured included scores on both the ACE tool and Fresno test using descriptive statistics. Spearman's rho was used to determine the correlation between the ACE and Fresno scores for each student among the entire group. A subgroup analysis was performed to assess for correlations at more extreme data points.

**Results:**

The median scores on the ACE tool and Fresno test were 66.7% and 62.7%. There was no statistically significant correlation between the results of these two assessments (Spearman's rho 0.023, p = 0.774) in our population. The scores from the subgroup of advanced performers on the Fresno test showed a weak statistically significant positive correlation (p = 0.045) to advanced scores on the ACE tool. No other subgroups showed statistically significant correlation.

**Conclusions:**

In our population of U.S. medical students, the results of two known EBM assessment instruments do not correlate with one another. The assessments may differ in what categories of learning they measure or in generalizability or perhaps in what depth of understanding they test overall. Further study is needed to determine what each instrument is measuring and whether there is demonstrable variation across populations.

## PURPOSE

Evidence‐based medicine (EBM) is “the integration of best research evidence with clinical expertise and unique patient values and circumstances.”^1^ The practice of EBM usually requires the following five steps: (1) translating the uncertainties into answerable questions (asking), (2) searching for and retrieving evidence to address the questions (acquiring), (3) critically appraising the evidence for validity and clinical importance (appraising), (4) applying the appraised evidence to inform the clinical decisions (applying or integrating), and (5) evaluating the performance in the previous four steps (assessing).^2^ It is understood that the final step is in effect a measure of improved outcomes based on actual clinical application and is therefore generally not assessed as part of the mission of EBM educational initiatives.

In undergraduate and graduate medical education, education in EBM is required. The Accreditation Council of Graduate Medical Education (ACGME) Common Program Requirements lists education in the appraisal of medical evidence as a core didactic activity and as a necessary skill in demonstrating practice‐based learning and improvement.^3^ The American Association of Medical Colleges (AAMC) lists the ability to appraise and apply evidence as a way of demonstrating competency in Core Entrustable Professional Activities (EPA) #7: the ability to ask clinical questions to advance patient care.^4^ However, as with much of the skill model of medicine, a clear and agreed‐upon representation of all the skills and behaviors that comprise EBM has proved elusive. The 2011 Sicily statement on classification and development of evidence‐based practice learning assessment tools put forward the CREATE framework, a rubric for EBM tools.^5^ That group, among others, acknowledge that rarely are trainees taught or assessed on achieved EBM competency on all steps in the EBM process.^5^, ^6^, ^7^

Attempts to demonstrate internal validity of several tools' scores have been made, but few have reported on practical differences between tools or compared results in the same population.^8^, ^9^ One of these tests, the Fresno test, was developed in 2003 with the goal of evaluating proficiency of EBM through the use of open‐ended questions to show higher order thinking.^8^ The goal was also to assess the learner in multiple areas of proficiency aside from just self‐reported improvement or critical appraisal alone.^8^ In this test, learners are presented with a clinical scenario and answer a series of twelve open‐ended free‐response questions.^8^ The best possible score is 212 points. Validation of the test was determined with administration among family medicine residents and self‐reported EBM experts to identify appropriate cutoff scores for the “novice in EBM” versus the expert taking the assessment.^8^ When comparing the initial dataset from which the Fresno test was developed to a dataset used for validation of the test, there was good inter‐rater reliability.^8^ The Fresno test assesses three of the five EBM steps set forth by the Sicily statement described above (ask, acquire, and appraise).^5^, ^8^ It is graded with a detailed rubric with each question broken down into multiple parts. Lists are provided of content that the tester must include to achieve different levels of scoring (absent, limited, strong, excellent) and points are assigned based on these varying levels. This rubric limits what is left to interpretation by the grader, but some level of subjectively inherently exists.^8^

The Berlin questionnaire is another EBM assessment tool that, like the Fresno test, was developed more than 15 years ago in 2002 and only assesses three of the five EBM steps.^9^ It differs from the Fresno test in that it is multiple choice and designed to follow a brief 3‐day EBM course.^9^ Reports of the Fresno test and Berlin questionnaire as assessments in undergraduate medical education number less than a dozen.^10^, ^11^, ^12^, ^13^, ^14^, ^15^, ^16^, ^17^ In 2011, West et al.^12^ applied both the Berlin and the Fresno assessments together, but an in‐depth comparison between the two was not reported. Lai et al.^14^ showed no correlation between the Berlin questionnaire and adapted Fresno test.

More recently, the ACE (Assessing Competency in Evidence Based Medicine) tool was developed in 2014, a significantly simpler test for measuring EBM knowledge of medical undergraduates built off a variety of prior EBM assessments by five experts.^15^ A cross‐sectional study was performed among 342 medical students in Australia with varying levels of training in EBM.^15^ The students were divided into cohorts based on this level of training. The variance and means of scores on the ACE tool were analyzed and the test was determined by authors to be valid and reliable.^15^ The authors argue that the strength of this new assessment tool is the ability to evaluate an additional step of the EBM process (application of results).^15^ In the ACE tool, learners are presented with a clinical scenario and answer 15 yes‐or‐no questions.^15^ The best possible score is 15.

Buljan et al.^18^ subsequently used all three of these instruments, the Fresno test, Berlin questionnaire, and ACE tool, in a population of third‐year medical students who took a 1‐week course, comparing that cohort's scores to those of one who had not received the intervention. The investigators primarily assessed the postintervention change in scores within each of the three assessments, without focusing on whether the different tools' results correlated with one another.

The Berlin questionnaire was not included in this study because the Fresno and the ACE were more readily available, and adding a third assessment was felt to increase the burden on students while providing no clear benefit to them. To obtain a copy of the Berlin questionnaire also often requires direct request from the authors who developed the tool making it an unfeasible option for many. Additionally, as the ACE tool is extremely brief and easy to grade, while the Fresno test takes significantly more time for learners to take and requires significant time from knowledgeable faculty to grade, the educators felt it would be important for efficiency if equivalence of the two instruments in this population was demonstrated. Thus, we sought to evaluate whether scores on two of the assessments with prior work demonstrating internal validity, the ACE and Fresno, correlated with one another in a population of U.S. medical students in their mandatory emergency medicine clerkship.

## METHODS

### Study design

This study was approved by our institutional review board (Pro2018001988) by noncommittee review for retrospective educational research. It is a retrospective cohort study examining whether a correlation exists between two EBM assessment tools on which prior validation studies have been performed, the ACE tool and Fresno test, in fourth‐year medical students exposed to an EBM curriculum. Both assessments were given to each student, and scores were analyzed individually. Three authors of this paper (AK, AS, SM) developed and implemented the curriculum in academic year 2018–2019 for fourth‐year medical students during their mandatory emergency medicine clerkship. Over the course of the 4‐week clerkship, medical students reviewed three different articles and participated in three moderated journal club–style discussions led by emergency medicine faculty and residents. Each conversation was designed to cover aspects of all four assessable steps of EBM.

The articles^19^, ^20^, ^21^ pertained to common emergency medicine topics: endovascular therapy for treatment of ischemic stroke, utilization of the HEART score for the management of chest pain, and the incidence of contrast‐induced nephropathy. They are, respectively, a systematic review and meta‐analysis, a randomized controlled trial, and a retrospective study. The same articles were used for each group of medical students. Over the course of the three sessions, the students are taught the first four steps of EBM. Students were provided critical appraisal worksheets from The Center for Evidence‐Based Medicine and the Critical Appraisal Skills Program to complete prior to the journal club discussion.^22^, ^23^, ^24^, ^25^ The authors served as facilitators during these sessions. They were aided by a facilitator guide created for each article by one of the study authors (AS) as a means of standardizing teaching across sessions (Appendix S1A‐C). The discussion questions on these facilitated guides were developed to address the four assessable stems of EBM as described above. There was no formal training given to the facilitators beforehand, and there was no other EBM teaching during the 4‐week clerkship. The authors were not blinded to study outcomes.

### Participants

All 158 students enrolled in the required emergency medicine clerkship at our 4‐year U.S. allopathic medical school were included in the study, from with data collection from June 2018 through May 2019. There were 11–18 students per clerkship block. There were no exclusion criteria. All of the students had participated in the same introductory biostatistics course during their first year of medical school. Many required third‐year clerkships have some information regarding EBM as a standardized part of their curriculum. As ours was a fourth‐year clerkship, all students would have completed the same EBM instruction in those courses. We did not survey students on elective or pre–medical school exposure to EBM curricula or information.

### Assessment administration

At the end of each clerkship block, students completed both the ACE and the Fresno assessments, which were administered together online via Qualtrics (QualtricsXM) as a single examination. The students were given a total of 75 min to complete the exam, having been told that their score would represent 5% of their total clerkship grade.

### Grading process

All assessments were divided and graded by one of two emergency medicine faculty, AK or AS. The ACE assessments were graded following the answer key provided by the original study. The Fresno assessments were graded according to the answer rubric provided by the original study, which includes a point‐by‐point breakdown of acceptable answers for each question. The time it took to grade each EBM exam type was not measured.

### Statistical analysis

All the data available from the cohort of this study were used; therefore, no sample size calculation was done. The volume and pace of grading of the Fresno test made calculation of inter‐rater reliability impossible with the resources available. All data were summarized and reported using descriptive statistics such as medians, means, and ranges. Data were tested for normality using the Shapiro–Wilk test. Spearman's rho was used to determine the correlation between the ACE and the Fresno scores for each student among the entire group. A subgroup analysis of both high‐ and low‐scoring students was also done to assess for correlations at more extreme data points. The defined cutoffs for the subgroups were chosen based on approximate values found in the original studies. In the ACE tool validation study, novice, intermediate, and advanced test‐takers corresponded to trainees with two, three, and four years of EBM training per the published report, who scored means of 8.6 (57.3%), 9.5 (63.3%), and 10.4 (69.3%), respectively.^15^ For the Fresno test, test‐takers categorized as novice represented a cohort of resident physicians who scored a mean of 95.6 (45%) while those considered advanced test‐takers were attending physicians, who scored a mean of 147.5 (69.6%).^18^ Since the difference between novice and advanced on the ACE tool was fewer than two points, and scores were reported as whole numbers, it was decided to assign a score of 8 or lower as the novice threshold and 11 or higher as the advanced threshold to create more separation between categories for the purposes of this analysis. As a result, the Fresno novice threshold was adjusted down to 90 and the advanced threshold was adjusted up to 150. All p‐values were reported at a significance level of 0.05. Figures were generated using Microsoft Excel.

## RESULTS

**FIGURE 1:**
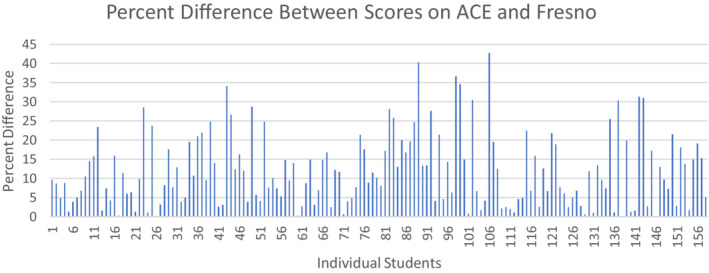
Correlation Between ACE and Fresno scores. Spearman's rho 0.023, p = 0.774

A total of 158 of 158 eligible fourth‐year medical students were included in our analysis. Scores on the ACE tool did not have a normal distribution in our population. The distribution of scores on the Fresno test was normal. On the ACE tool, the median score in this population was 10 out of a possible score of 15 (66.7%). The median score on the Fresno test was 133 out of a possible 212 (62.7%). There was no statistically significant correlation between the ACE and Fresno scores in the entire cohort of students (Spearman's rho 0.023, p = 0.774; Figure 1).

The results of the subgroup analysis for different levels of test‐taker performance are shown in Table 1. For students who scored at an advanced level on the ACE tool there was no statistically significant correlation to achieving an advanced level on the Fresno test. For advanced scores on the Fresno test, there was a weak, statistically significant positive correlation to advanced scores on the ACE tool. For novice scores, the ACE tool and Fresno test did not correlate with each other.

**TABLE 1:** Correlation between ACE and Fresno scores for novice and advanced performers

Independent variable	Dependent variable	Spearman's rho	p‐value
11+ ACE (advanced)	Fresno	0.128	0.317
150+ Fresno (advanced)	ACE	0.369	0.045
8– ACE (novice)	Fresno	−0.155	0.69
90– Fresno (novice)	ACE	0.387	0.391

We also compare the average percent score of students in our study cohort who answered each question correctly to the original ACE and Fresno validation study cohorts (Table 2). Notably there was wide variability in scores per question.

**TABLE 2:** Comparison of average % correct between our study cohort and the ACE and Fresno validation cohorts

*A. ACE tool*		
Population	Monash University, Australia	U.S. Medical School
	342 medical trainees in Melbourne, Australia	158 fourth‐year medical students in Newark, New Jersey, USA
Average % correct		
Q1	69	80
Q2	74	52
Q3	84	94
Q4	60	95
Q5	70	74
Q6	43	4
Q7	49	54
Q8	66	100
Q9	81	98
Q10	66	92
Q11	59	30
Q12	79	87
Q13	76	97
Q14	40	55
Q15	36	21

*B. Fresno test*			
Population	University of California San Francisco Fresno		Allopathic Medical School
	43 family medicine residents and faculty in San Francisco, California		158 4th year medical students in Newark, New Jersey, USA
Average % correct	Novice	Expert	
Q1	27	80	81
Q2	61	75	63
Q3	17	64	72
Q4	44	80	62
Q5	30	37	62
Q6	56	91	61
Q7	12	58	54
Q8^a^			98
Sensitivity	60	84	
Specificity	33	76	
Positive predictive value	40	71	
Negative predictive value	35	66	
Likelihood ratio positive	15	58	
Q9^a^			72
Absolute risk reduction	33	87	
Relative risk reduction	10	76	
Number needed to treat	30	87	
Q10	26	86	82
Q11	10	39	21
Q12	41	83	70

^a^ Questions 8 and 9 on the Fresno assessment are multipart questions. While the original Fresno study reported the average score for each part, our study collected the average score for the entire question only.

## DISCUSSION

Scores on two validated EBM assessment tools do not correlate with one another in our population of senior medical students. The many structural differences between the tools could explain this finding: the two assessments are scored on different scales, with their own scoring ranges due to their inherently different testing and grading formats. In addition, the cohorts in the ACE and Fresno validation studies were somewhat different from one another (trainees in the Australian medical system versus family practice residents in the western United States, respectively) and are both different from our cohort of fourth‐year medical students in the northeastern United States.

Given that the ACE tool is true/false and quite brief, while the Fresno test requires short answers created de novo by each learner, there may be important psychometric reasons for the discrepancy we found, which might be more demonstrated at lower skill levels. This idea is weakly supported by the statistically significant correlation we found between scoring highly on the Fresno test and on the ACE tool. The Fresno test also requires some interpretation on the part of the graders, which could lead to measuring error in one but not the other. It is also possible that our population's prior education or our curriculum favors one assessment disproportionately. However, our students overall achieved just below expert and advanced levels on the ACE and Fresno exams, respectively, suggesting competency on both assessments.

To date, most studies using the ACE, Fresno, or Berlin assessments have done so to gauge the effectiveness of a curriculum. In a recent systematic review of teaching EBM to medical students, 27 studies were identified. The majority were nonrandomized, contained a high risk of bias, and did not evaluate long‐term effects. Eleven of these studies examined the effects of seminars, workshops, and short courses, of which eight found positive effects.^26^ Also of note, there are questions—most notably numbers 6 and 11 on the ACE tool and 1, 5, and 10 on the Fresno test—in which our population performed markedly better or worse than would be expected based on the prior validation studies. Thus, it remains difficult to tell if the instruments are good metrics for growth; which tool is a more accurate measure of skill within a particular system, population, or level of expertise; or whether they in fact are testing the same skills. Additionally, we did not examine test results for the Berlin questionnaire, the third known validated EBM assessment. It is possible this exam could correlate to both the Fresno and the ACE and be the best overall examination.

It is possible that, in some medical systems or levels of training, the Fresno test is a better overall assessment than the ACE tool, and vice versa, although more work would need to be done to externally validate each tool in our population of medical students. Our study was limited to discovering correlation, or lack thereof, between the assessments. In our cohort, there appears to be no clear‐cut superior choice, but it bears noting that, for a team with limited resources, the ACE tool is significantly easier to implement. Whereas for an educational team hoping to develop their own understanding of the ways in which EBM can be understood, taught, and assessed, the Fresno test—with its more granular approach and more qualitative grading rubric—might be a better choice. More work on what skill set or knowledge is demonstrated with each assessment, and test item, might help educators more usefully tailor and compare curricula.

## LIMITATIONS

There are important limitations to our study. First, this was not a randomized or blinded study, so our results could be subject to bias on the part of the curriculum facilitator and grader as well as unmeasured confounders. In particular, the Fresno test is subject to some qualitative differences in interpretation and grading despite its clear grading rubric. Given the labor involved in grading such a test, we split the work among the faculty, and there may have been variability in scoring. The time required made redundant grading and thereby calculating inter‐rater reliability impracticable.

Our cohort consisted of fourth‐year medical students in the United States, which is different from the studied cohorts in the ACE and Fresno validation studies. This makes the interpretation of test performance challenging, as our results may not apply to students of different learner levels or in other medical training systems. Furthermore, limitations to use of both tests is that they are available via open access. There is a slight chance that a learner may have some prior knowledge. Additionally, psychometric reasons could potentially account for the poor correlation observed. More research would be required to evaluate this.

## CONCLUSIONS

Scores on two validated tests for evidence‐based medicine assessment do not correlate in our population of fourth‐year medical students. Further study is needed to determine which skills are demonstrated with success on each tool, the reliability of the tests, the discrimination power of each item, and which assessment is best in a particular setting.

## AUTHOR CONTRIBUTIONS

Adam Kenney conceived of the project, administered much of the teaching and grading, performed statistical analyses, and wrote the majority of the manuscript. Catherine Yu assisted with both the administration and the actual teaching of the EBM curriculum and assisted with statistical analysis, figure creation, and manuscript writing and editing. Ariel Sena assisted with teaching and grading for the EBM curriculum, created the facilitator guides upon which the curriculum is based, graded assessments and assisted with editing and analysis of the data collected, and wrote the original proposal and IRB approval. Naila Ghafoor assisted in the final stages of editing and formatting the manuscript as well as working to resolve some statistical analysis and focus questions. Shannon Moffett was the director of the course in which this curriculum was implemented; assured approval at the school and department level of the curriculum; ensured compliance with IRB requirements for research with students; and assisted in the conception, administration, logistics, data collection, and analysis at all points in the initiation, development, and completion of this project.

## CONFLICT OF INTEREST

The authors declare no potential conflict of interest.

## Supporting information


Appendix S1
