# Supplementary material for: Comparison of two validated evidence‐based medicine assessments: Do they correlate?
Source: AEM Educ Train. 2022 Dec 20;6(6):e10831. doi: 10.1002/aet2.10831 (PMC9764041; doi:10.1002/aet2.10831)
Supplement: Supplementary file 1 — Appendix S1 [file AET2-6-e10831-s001.docx]

1. Facilitator’s Guide to Endovascular Intervention in Stroke

To accompany the following article:

Rodrigues FB, Neves JB, Caldeira D, Ferro JM, Ferreira JJ, Costa J. Endovascular treatment versus medical care alone for ischaemic stroke: systematic review and meta-analysis. *Bmj.* 2016;353:i1754.

*Instructions for facilitators: The following are questions to present to students to stimulate discussion regarding the articles. Below some of the questions are responses in italicized to help you answer the posed questions with the group.*

- 1. Introduction
  2. What are the different types of stroke?
     1. *Hemorrhagic* vs *Ischemic*
  3. Introduce what happens when a patient suspected of acute stroke presents in our ED, what is the process?
     1. *Will vary based on institution - Comprehensive stroke* *center, the importance of the fast CT, assess the criteria for tPA (do not get into discussion about tPA)*
  4. 2^nd^ paragraph – They discuss the improvement or success of treatment as measured by both recanalization rates and functional outcome. What’s the difference between these outcomes? Which is more important? What should we be measuring?
     1. *Highlight that a recanalization rate is an example of a disease* *centered outcome* *while assessment of functional outcome is a patient* *centered outcome. Define disease* *centered* vs *patient* *centered outcomes. Emphasize that patient* *centered outcomes are usually more desirable for studies.*
  5. What is thrombectomy? What does it add to the treatment?
     1. *Ensure that students understand what happens with thrombectomy for acute stroke and what it adds to the treatment.*
  6. Briefly describe heterogeneity and what it means (last paragraph before “methods”): the trials discussed in the intro were very different and difficult to compare.
  7. Do you think this was an important study to do? Why?
     1. *Emphasize with students - Why do we need this study? Use the literature cited by authors in introduction to answer this question.*
  8. What question did this study ask? (PICO)

**P**opulation – adults with ischemic stroke

**I**ntervention – endovascular treatment

**C**ontrol – standard medical care (tPA)

**O**utcome – Primary: efficacy; Secondary: safety

- 1. Methods
  2. What type of study is this?
     1. *Meta-analysis – define for students as needed.*
  3. What are the inclusion and exclusion criteria? Why do we care about what the criteria are when we are looking at a study?
     1. *Look for any groups that were excluded and could be source of bias. Also look to see if the patients included in the study are similar to our own patient population and informs us about generalizability.*
  4. Where did they look to obtain studies and data?
     1. *It is important in a meta-analysis to include ongoing clinical trials and MESH terms. (It is unclear if MESH terms were used in this case.)*
     2. *Try to discuss how to do a search for articles. What are keywords* versus *MeSH terms?*
     3. *Note that it is important to include studies not in English.*
  5. How did they choose which studies to review?
     1. *Describe the two reviewer process and how the authors ensured the two reviewers agreed.*
  6. What is intention to treat?
     1. *When performing the statistical analysis, patients are analyzed as part of the treatment arm to which they were originally assigned. This is done even if the patient stopped the assigned treatment or initiated another treatment.*
     2. What type of analysis is typically preferred? Intention to treat or per protocol?
        1. *Intention to treat is usually preferred to help reduce bias. The following example can be used to help explain this to the students. –*

*In a study comparing drug A to drug B, patient x was assigned drug A. This patient stopped taking drug A and began taking drug B. Intention to treat analysis would keep patient x in the drug A group (the group to which he was originally assigned). We have to ask why did patient x stop taking drug A? Was it a negative side effect of drug A? If we analyze patient x in the drug B group, this can skew the data and potentially reduce the ability to identify the potential harms of drug A.*

- 1. What was the primary outcome?
     1. *Efficacy outcome: Proportion of patients achieving modified rankin score of 0-2 at 90 days*
     2. *Safety outcome: All-cause mortality at 90 days*
  2. What is modified Rankin Score?
     1. *This is a standardized scoring system to rate a patient’s neurological functional status.*
     2. *0 is no symptoms, 6 is dead*
     3. *2 is slight disability, cannot do everything could do before, but can look after own affairs without assistance*
  3. How did the authors consider bias in each study?
     1. *The authors used the Cochrane Collaboration risk of bias tool, a standardized method of assessing bias.*
     2. *They also included three additional criteria: independent funding, early stopping of trial, and clinical trial registration.*
     3. *Registering a trial prospectively helps reduce reporting bias.*
     4. *It is important to also consider publication bias. There may be studies that were conducted but results were never reported (usually because they are unfavorable or show no difference).*
  4. What is type I and type II error?
     1. *Type I error: rejection of the null hypothesis when it is actually true (false positive)*
     2. *Type II error: failure to reject the null hypothesis when it is actually false (false negative)*
  5. Why do you think the authors decided to look at the data separately for 2013 and 2015 trials?
     1. *This is perhaps due to the availability of new devices for the 2015 studies. New devices could affect the safety and efficacy.*
     2. *Studies performed in 2015 additionally required radiological evidence of a lesion (which is consistent with how we* *practice at our institution).*
  6. Results
  7. Figure 1: This describes the process that the authors used to select the studies included in this systematic review and meta-analysis. Do you agree with the methods and think that this was appropriate?
  8. Cohen’s κ coefficient – a value greater than 0.6 is considered good; The κ in this study was 0.75. This measures how good the interobserver agreement is.
     1. (*We did not expect our students to know the definition of Cohen’s κ coefficient. We simply pointed it out to them and briefly explained it as described above.)*
  9. Table 1: This describes the different characteristics of the studies. What observations do you make about these studies?
     1. *Highlight trial period and number of* *centers involved. This demonstrates how some studies included a very select patient population.*
     2. *Look at the variability in the NIHSS scores, also demonstrates how some were more select than others.*
     3. *Using the examples of IMS III and MR CLEAN highlight the above points well.*
     4. *The characteristics of the studies contributes to our ability to apply these data to our patient population.*
  10. Where was the location of the lesion in the patients?
      1. *Important to note that all studies looked at thromboses in the anterior circulation. This limits our ability to apply this study to strokes in posterior circulation.*
  11. Table 2 – This table informs us about the characteristics of the patients included and how they compare from study to study.
  12. Table 3: This table informs us about the characteristics of patients included in each treatment arm. Look at the number of patients assigned to a given arm and the number that actually received the assigned treatment. Why might this be a problem?
      1. *Students should note that there as a poor adherence rate to the protocol. For example, in the SYNTHESIS trial, only 30.9% assigned to thrombectomy actually received this therapy, compared to the ESCAPE trial where 91.5% assigned to thrombectomy received the therapy. Discuss some of the problems with lack of adherence to the protocol. Is the study actually measuring what it intends to measure?*
  13. Ask a student to interpret Figure 2.
      1. (+) in the figure indicates that this type of bias was not present in the study, so it is a strength of that particular study.
      2. Review the different types of bias with the students. Highlight the last three columns (these were the three additional criteria authors chose to include).
         1. *Independent Funding – Many studies do not include if there was independent funding. A company potentially funding and influence the research for their own product or treatment creates a large risk of bias.*
         2. *Trial Stopped Early – Without including the intended number of participants, we are unsure if the results are simply due to chance.*
         3. *Prospective clinical trial registration – Trials should be registered before data collection begins. It helps inform us if there were multiple studies conducted on a topic, but maybe not published because of unfavorable results.*
  14. Figures 3 and 4 – Break down how to interpret a forest plot.
      1. *Figure 3 is demonstrating the results for the outcome of efficacy. Figure 4 shows the results of mortality.*
      2. *Black Square = risk ratio (relative risk in exposed group/relative risk in unexposed group) for the particular study.*
      3. *Diamond = collective risk ratio of all studies included. The authors broke down the study into two subgroups as well, one for the 2013 studies and one for the 2015 studies.*
      4. *Vertical Line = 1*
      5. *Horizontal Line = Confidence interval. If the horizontal line (confidence interval) crosses the vertical line (1), then the results of that study are not significant.*
      6. *Size of the shape (large square* vs *small square) represents the weight of the study.*
      7. *The authors also report the heterogeneity, or how similar the studies were.* I.e. *Are you comparing apples to apples? Or are you comparing apples to oranges? I^2^ is one way to measure this. 0% is best. 100% is worst. >50% is considered poor or a high amount of heterogeneity.*
      8. *Figure 3 favors endovascular treatment and is overall statistically significant.*
      9. *Figure 4 trends* *towards favoring medical care only, but it is not statistically significant.*
  15. What is the meaning of a risk ratio? How do you explain what the number of a risk ratio means? Interpret the meaning of a risk ratio of 1.37 (middle of the page on page 7). “Patients receiving endovascular treatment had a higher chance of achieving a good outcome (risk ratio 1.37, 95% confidence interval 1.14 to 1.64; fig3)…”
      1. *This can be interpretated as: Patients receiving endovascular treatment had a 1.37 times increased risk of achieving a good outcome, or they had a 37% increased risk of achieving a good outcome compared to patients who did not receive endovascular treatment.*
  16. With treatment for stroke (both with intravenous tissue plasminogen activator and endovascular intervention), we worry about the risk of intracerebral hemorrhage to the patient. It is therefore important to note the rate of intracerebral hemorrhage in this study which authors report as 5.1%.
  17. Discussion
  18. What do the authors describe were the limitations of the study?
      1. Not powered enough to evaluate the safety of endovascular treatment
      2. Observational studies may be more adequate than RCTs to evaluate safety as they may include patients usually excluded from RCTs.
      3. Magnitude of effects may have been exaggerated in the studies due to selective patient inclusion.
      4. Open blinded studies may lead to more bias from researchers and participants.
      5. A follow up of 90 days may not be long enough to evaluate patients with spontaneous neurological recovery.
      6. All trials had an overall moderate risk of bias.
  19. What does this add to the literature?
  20. What do you think of the results? Would this change your clinical practice?
  21. What treatments would you recommend to a patient with a thrombotic stroke in the anterior circulation?
  22. How will you explain the risks and benefits to a patient when discussing this possible treatment with a patient?

1. Facilitator’s Guide to The Heart Pathway

To accompany the following article:

Mahler SA, Riley RF, Hiestand BC, et al. The HEART Pathway randomized trial: identifying emergency department patients with acute chest pain for early discharge. *Circulation Cardiovascular quality and outcomes.* 2015;8(2):195-203.

*Instructions for facilitators: The following are questions to present to students to stimulate discussion regarding the articles. Below some of the questions are responses in italicized to help you answer the posed questions with the group.*

- 1. Background
  2. Why is this study important?
     1. We see an enormous volume of complaints of chest pain in the ED and think about ACS many times throughout a shift.
     2. Acute Coronary Syndrome (ACS) workups with the usual care are very costly.
     3. Current guidelines do not accurately focus resources on the individuals who are most likely to benefit. In patients with low risk chest pain, “…objective cardiac testing is associated with a substantial number of false-positive and nondiagnostic tests, which often lead to invasive testing.”
     4. There is a need to evaluate these patients in a more efficient but safe manner.
  3. What is the HEART score? What are the components?
  4. Summarize the HEART Pathway using Figure 1.
  5. Last paragraph: “Observational studies have demonstrated that the HEART Pathway can classify >20% of patients with acute chest pain for early discharge while maintaining a negative predictive value (NPV) for MACE rate >99% at 30 days.” What is NPV?
     1. Maintaining a NPV of >99% means that, given a negative test result, the probability that the disease will be absent is >99%.
  6. What question did the study ask? (What are the elements of PICO?)

*Population – Patients older than 21 yo with symptoms suggestive of ACS*

*Intervention –The HEART Pathway (HEART score + 0 and 3 hour troponins)*

*Comparison –Usual care: defined by the American College of Cardiology Guidelines (serial troponins + objective cardiac testing)*

*Outcome – Primary outcome: objective cardiac testing;*

*Secondary outcome: index length of stay, early discharge, Major Adverse Cardiac Event (MACE) at 30 days*

- 1. Methods
  2. What is the study design?
     1. *Single* *center randomized controlled trial*
  3. What is the study setting? Where did the study take place?
     1. *It is important to identify if the setting is similar to our own institution to consider its generalizability to our patients.*
  4. Discuss the inclusion and exclusion criteria
     1. *Note the non-English speaking individuals were excluded from the trial* - Why is this important? *(Do non-English speakers have a higher risk of ACS? Leaving out entire group of people can contribute to confounding.)*
  5. Was the assignment of patients to treatments randomized?
     1. *Yes, patients were stratified based on risk and then randomized.*
     2. *Providers were blinded to the random sequence.*
  6. We should note that the participants in the control group were *encouraged*, but not required to follow American College of Cardiology Guidelines, there was no protocol. Why is this good or bad?
     1. *Traditionally, in trials, the control arm should adhere to a designated protocol. Practically, clinicians do not always adhere to guidelines. By encouraging the control group to adhere to guidelines, this may more accurately reflect real life. It may better reflect how a clinical decision tool would actually be used. It could be argued that this improves the validity of the heart pathway.*
  7. What is interobserver agreement? Why is it important?
     1. *If multiple clinicians are using the same clinical decision tool, we want to know if they get the same result or score for an individual patient.*
  8. How did the authors conduct follow up?
     1. *At the index visit, there was a structured record review.*
     2. *At 30 days, there was a second structured record review, followed by a telephone interview for any missing information. Authors also performed a structured record review of medical records at other healthcare facilities. The Social Security Death Master File was also used to search for any patients lost to follow up.*
  9. Do you think this follow up was complete and a good way of conducting follow up?
  10. Outcomes
  11. What were the primary and secondary outcomes?
      1. *Primary: Rate of objective cardiac testing within 30 days of presentation*
      2. *Secondary: Early discharge rate, Index length of stay (LOS), Cardiac-related recurrent emergency department (ED) visits, and non-index hospitalization at 30 days*
  12. What were the safety events?
      1. *All were monitored for* *MACE. MACE defined as composite end-point of: all cause mortality, myocardial infarction, or coronary revascularization within 30 days.*
  13. What is a composite endpoint?
      1. *A composite endpoint combines multiple outcomes or variables of interest into one. It is usually used when events of interest are rare.*
  14. Do you think that all outcomes are equal? Should they be equal?
      1. *One could argue that a patient suffering from death is very different than a patient requiring coronary revascularization and then proceeding with a nearly normal functioning life. A composite endpoint can be a problem because it does not distinguish for the reader what actually happened to these patients suffering from “MACE”.*
  15. Results
  16. Look at figure 2 – What type of analysis was used to assess the data? Why is this important?
      1. *Intention to treat analysis – All patients who were enrolled and randomly allocated to the two study arms are included in the analysis and analyzed in the group to which they were assigned regardless of any deviations that may happen after randomization.* i.e. *If a patient was randomized to the HEART Pathway arm, but actually received usual care, the patient was included in the HEART Pathway group for analysis.*
      2. *This provides a more reliable estimate of the true treatment effect by replicating what happens in the real world.*
      3. *This helps to guard against conscious or unconscious attempts to influence results of the study by excluding odd outcomes. It helps to prevent bias when incomplete data is related to an outcome. It also helps preserve the baseline balance between the groups.*
      4. *It minimizes type I error (false positives).*
  17. Look at Table 1: Do you think the groups were equal?
      1. *These types of tables are common in randomized controlled trials (RCTs). It helps to show the reader whether or not the groups are equal at the beginning of the study.*
  18. Table 2 demonstrates the importance of obtaining a 3 hour troponin in addition to the initial troponin. 6.4% would otherwise be missed.
  19. What were the results for the primary and secondary outcomes? (Tables 3 and 4)
      1. *Primary Outcome: Rate of objective cardiac testing at 30 days – 68.8% in the usual care group and 57.7% in the HEART Pathway group. This is an absolute reduction of 12.1% (p = 0.048)*
      2. *Secondary Outcomes:*
         1. *Early discharge – 18.4% in the usual care group and 39.7% in the HEART Pathway group. This is an absolute increase by 21.8% (p < 0.001);*
         2. *Length of stay – mean reduction of 12 hours (p = 0.013)*
      3. *Neither group missed any MACE in 30 days.*
  20. Are these results statistically significant? Are they clinically significant?
      1. *P values inform you if a result is statistically significant,* i.e. *we can be fairly confident that the results are not due to chance.*
      2. *To determine if something is clinically significant, we must determine this ourselves as clinicians. Number needed to treat and number needed to harm can help inform this decision.*
  21. What is the number needed to treat?
      1. *NNT = 1/ absolute reduction*
      2. *Rate of 30 day objective cardiac testing – NNT = 1/12.1% =* ***8***
      3. *Early discharge – NNT = 1/21.3% =* ***5***
  22. Look at the non-adherence rates (in last couple of sentences of results section). What would have happened to early discharge if there was perfect adherence?
      1. *“Perfect adherence* *among high- and low-risk patients would have increased the early discharge rate to 46.8% (66/141).”*
  23. Figure 3 demonstrates Kaplan Meier Curves for length of stay. This visually shows the difference between the usual care and HEART Pathway arms. We will further discuss Kaplan Meier Curves when we review the next article (Contrast Nephropathy).
  24. Table 5 - What are the definitions of sensitivity, specificity, positive predictive value (PPV), negative predictive value (NPV)? What do we care about in this case and why?
      1. *Sensitivity and specificity are characteristics of the test. PPV and NPV are characteristics of the patient.*
      2. *Sensitivity: The probability that a person with the disease will test positive. A high sensitivity is helpful to rule out a disease. Sensitivity = (True Positives)/(True Positives + False Negatives)*
      3. *Specificity: The probability that a person without the disease will test negative. A high specificity is helpful to rule in a disease. Specificity = (True Negatives)/(True Negatives + False Positives)*
      4. *Positive Predictive Value (PPV): The probability that a patient truly has a disease given a positive test result. PPV = (True Positives)/(True Positives + False Positives)*
      5. *Negative Predictive Value (NPV): The probability that a patient truly does not have a disease given a negative test result. NPV = (True Negatives)/(True Negatives + False Negatives)*
      6. *The authors describe in the background that they sought “to determine whether the HEART Pathway can meaningfully reduce objective cardiac testing, increase early discharges, and reduce index hospital length of stay (LOS) compared with usual care* *while maintaining high sensitivity and NPV (>99%) for MACE.” We want to be able to use this clinical decision tool and be sure that the patients we are discharging truly do not have ACS and will not have MACE within 30 days.*
  25. Discussion
  26. What are some of the strengths of this study?
      1. *The authors did not enforce strict adherence to the study protocols. This may better resemble the way a clinical decision tool would be used in real life. It therefore arguably better demonstrates its real world effect.*
      2. *Intention to treat analysis*
      3. *Little loss to follow up*
      4. *Clinically meaningful results.*
  27. What are some of the limitations of this study?
      1. This study was done only in ED patients. It cannot be applied to inpatients. It is important to consider patient populations when making decisions to use clinical decision tools.
      2. Small sample size may limit generalizability.
  28. Based on the results of this study, would you feel comfortably implementing this into your clinical practice? Why or why not?

1. Facilitator’s Guide to Risk of Acute Kidney Injury after IV Contrast

To accompany the following article:

Hinson JS, Ehmann MR, Fine DM, et al. Risk of Acute Kidney Injury After Intravenous Contrast Media Administration. *Ann Emerg Med.* 2017;69(5):577-586.e574.

*Instructions for facilitators: The following are questions to present to students to stimulate discussion regarding the articles. Below some of the questions are responses in italicized to help you answer the posed questions with the group.*

- 1. Background
  2. Have you been on a rotation when you wanted to order a contrast CT study but did not because you were worried about acute kidney injury (AKI)? In what scenarios did this occur?
  3. Why is this study important? Why do the authors argue it is needed?
     1. *Our knowledge on this topic comes from studies with a number of problems that limit their applicability to our* *practice today.*
        1. *Prior studies included analysis from arterial contrast as opposed to intravenous (IV) contrast only.*
        2. *The contrast that was used in older studies contained a higher osmolarity than the commonly used contrast material today.*
        3. *The prior studies were observational and any of them lack control/comparison groups. It was considered unethical to perform a randomized controlled trial (RCT) because it was accepted that contrast material is a direct cause of kidney injury.*
        4. *These prior studies come to different conclusions.*
  4. Because the prior studies were observational, the authors argue that past studies are severely limited by selection bias. What is selection bias?
     1. *Participants in a trial are systematically different than the population of interest. Lack of randomization leaves studies prone to selection bias.*
  5. What is the study question? (PICO)

***P****opulation – adults that presented to the ED and had serial measurements of their creatinine*

***I****ntervention – contrast enhanced CT*

***C****ontrol – non-contrast CT AND no CT (there are two control groups)*

***O****utcome – Primary outcome: acute kidney injury*

*Secondary outcome: development of CKD, need for hemodialysis, need for renal transplant*

- 1. Are these outcomes disease centered or patient centered?
     1. *AKI is a disease* *centered outcome. The secondary outcomes of development of CKD, need for hemodialysis and need for renal transplant are all patient* *centered outcomes.*
  2. Methods
  3. What is the study design?
     1. *Single* *center retrospective cohort study*
     2. *Multiple authors extracted the data (helps reduce bias).*
  4. What are the inclusion and exclusion criteria?
     1. *Refer to paragraph “Selection of Participants” on page 579.*
  5. Can you think of other chronic morbidities that could affect kidney function?
     1. *Inclusion criteria should be inclusive but also help us to isolate the intervention/study question.*
  6. Why did the authors include the group that did not get a CT?
     1. *The authors include this group in attempt to minimize bias associated with the decision to obtain imaging. It could be argued that patients who require a CT scan are already sicker and therefore at higher risk of developing AKI regardless of whether or not they obtain IV contrast material.*
  7. What difference did the authors think was significant difference to look for?
     1. *The authors report that they used a difference of at least 1.5% in rates of AKI between the groups to calculate their sample size. This should be a number that is sought out in every study you read. Where do authors obtain this number? It is best informed by prior research available on the subject, but sometimes this information is not available to the authors. You must ask yourself if you think a difference in rate of AKI of 1.5% is clinically meaningful to you and your* *practice.*
  8. What were the variables collected?
     1. The primary variable of interest was whether or not IV contrast material was administered.
     2. Secondary – age, sex, race… (see paragraph at top of page 579)
  9. It is also important to note whether or not an intervention is feasible at one’s own institution.
     1. *The type of contrast used in the study is the same that we administer at our institution, helping to verify that this study is relevant to us.*
  10. Part of their protocol required that all patients with a baseline creatinine >1.7 mg/dL sign consent to receive IV contrast. Why might this be a problem for the study?
      1. *This may have deterred providers from obtaining CT scans in patients with higher creatinine levels, even though the inclusion criteria states that patients with a creatinine of 0.4 to 4.0 were eligible. This could introduce bias into the results.*
  11. What was the primary outcome?
      1. The incidence of AKI (disease oriented)
  12. What was their definition of AKI?
      1. *Two definitions were used for AKI.*
         1. *The most frequently published criteria for contrast-induced nephropathy – absolute increase in serum creatinine level by at least 0.5 mg/dL or at least 25% increase over baseline serum creatinine at 48-72 hours after imaging, or for non-CT patients, after initial serum creatinine measurement.*
         2. *As defined by the Acute Kidney Injury Network/Kidney Disease Improving Global Outcomes guidelines.*
  13. What were the secondary outcomes?
      1. *Incidence of development of CKD, need for HD, renal transplant (patient* *centered)*

(*Facilitators can briefly define the following terms described in the primary data analysis section. We do not expect students to know the details about this nor believe it is important to get into a deep discussion about it.*)

- 1. Multi-logistic regression analysis was used to analyze the data.
     1. *This is used when there is one dependent variable and multiple independent variables. When you think that multiple independent variables could have an effect on the dependent variable. This test allows you to measure that effect.*
  2. Propensity score matching was also used in the analysis. This accounts for the covariates in attempt to see the true effect of the variable in question
     1. *This allows you to compare the effect of the intervention on groups that are not equal. Ideally in an RCT, we want to compare groups that are similar in all aspects except for the intervention in question. When we are unable to perform an RCT, and the groups we are comparing are different, we can use propensity score matching in attempt to isolate and understand the effect of the intervention*
  3. Results
  4. How were patients excluded? Was this reasonable? (Look at figure 1)
  5. Look at table 1. Were the groups similar? What are some characteristics that differ between the different groups?
     1. *ED critical care designation*
     2. *Nephrotoxic medications administered*
     3. *Crystalloid fluids*
  6. Table 2 informs us on the rates of AKI based on the different characteristics. It highlights the differences between these definitions and perhaps the problem with using disease centered outcomes.
  7. Looking at Table 3, what are the overall findings? (*We find it best to focus only on the two rows labeled “overall”. It can otherwise become very confusion and difficult for students to follow.)*
     1. CIN Criteria: The rates of AKI were very similar in the three groups. There was no statistically significant difference identified in incidence of AKI when comparing the groups. This was not affected by propensity score matching.
     2. AKIN Criteria: The group that had contrast enhanced CT had a lower rate of AKI compared to the two groups that had no IV contrast administration. The odds ratios for this comparison demonstrates that this difference is statistically significant. However, after propensity score matching is applied, this difference is no longer statistically significant.
  8. The authors report the probability of developing CKD within 6 months of the index visit as the following: 2.0% in the contrast enhanced CT group, 4.6% in the unenhanced CT group and 3.5% in the no CT group. Why do you think the unenhanced CT group had the highest rates of development of CKD and probabilities of initiation of dialysis?
     1. *Maybe the patients who underwent non-contrast CTs were very sick at baseline. The patient may have already been at increased risk to develop kidney injury. This is perhaps why the provider chose not to use IV contrast.*
     2. *This effect was revoked after propensity score matching.*
  9. Look at figure 2: What does this tell us?
     1. This shows the baseline creatinine in the patients who received contrast enhanced CTs. The majority of the patients had a creatinine less than 2.0. This informs us that while the inclusion criteria listed patients with a creatinine of 0.4-4.0, we have data mostly on patients with a creatinine of up to 2.0. We need to be careful about applying this data to patients with creatinine >2.0. This is perhaps related to the performance bias of requiring patients with a creatinine of 1.7 or higher to sign for consent prior to contrast administration.
  10. Discussion
  11. What are some limitations of this study?
      1. *Single academic* *center*
      2. *Only studied admitted patients – There are many patients that we order CTs, give contrast to and discharge. Admitted patients are arguably sicker and may have higher risk of developing AKI to begin with. Maybe rates of AKI would be even lower if included patients who are discharged.*
      3. *Retrospective observational study – We have to be careful about using results from a single observational study to inform our* *practice. Results from observational studies are helpful to allow us to generate hypotheses or support that a randomized controlled trial is in fact ethical to do. It is important to remember that for some clinical questions, we may never be able to perform a randomized controlled trial and collective data from multiple observational studies may be the best evidence we can get.*
